# Is there a duty to participate in digital epidemiology?

**DOI:** 10.1186/s40504-018-0074-1

**Published:** 2018-05-09

**Authors:** Brent Mittelstadt, Justus Benzler, Lukas Engelmann, Barbara Prainsack, Effy Vayena

**Affiliations:** 1 Oxford Internet Institute, University of Oxford, 1 St. Giles, Oxford, OX1 3JS UK; 2 Robert Koch Institute, Berlin, Germany; 3 University of Edinburgh, Edinburgh, UK; 4 University of Vienna, Vienna, Austria; 5 King’s College London, London, UK; 6 Universitat Zurich, Zurich, Switzerland

## Abstract

This paper poses the question of whether people have a duty to participate in digital epidemiology. While an implied duty to participate has been argued for in relation to biomedical research in general, digital epidemiology involves processing of non-medical, granular and proprietary data types that pose different risks to participants. We first describe traditional justifications for epidemiology that imply a duty to participate for the general public, which take account of the immediacy and plausibility of threats, and the identifiability of data. We then consider how these justifications translate to digital epidemiology, understood as an evolution of traditional epidemiology that includes personal and proprietary digital data alongside formal medical datasets. We consider the risks imposed by re-purposing such data for digital epidemiology and propose eight justificatory conditions that should be met in justifying a duty to participate for specific digital epidemiological studies. The conditions are then applied to three hypothetical cases involving usage of social media data for epidemiological purposes. We conclude with a list of questions to be considered in public negotiations of digital epidemiology, including the application of a duty to participate to third-party data controllers, and the important distinction between moral and legal obligations to participate in research.

## Introduction

In 2001, Ruth Chadwick and Kåre Berg asked whether a duty exists for the public to contribute samples and data to genetic databases. At that time, large-scale genetic databases that could be linked and mined were a relatively new development; they promised to generate powerful insights into links between health, treatment and genetics. At the same time they posed privacy risks and challenged existing models of informed consent. This was the case because “biobanks” used new ways of linking and mining data, and the very format of a bioresource escaped the assumptions of traditional research ethics where there was a specific group of patients affected and a clearly bounded intervention (Metcalf 2017). While some see the biobank bubble as having burst (Chalmers et al. 2016), a similar push for ‘Big Data’ is now underway in epidemiology. These promises and challenges are being pushed a step further: the proliferation of personal, digital data, so the argument goes, has created new opportunities for epidemiological research and public health interventions. Similar to what Chadwick and Berg argued in relation to genetic databases in the early 2000s, existing ethical frameworks may be insufficient to make sense of the ethical challenges posed by exploitation of digital, social data in epidemiology—what we refer to as ‘digital epidemiology’ (DE).

Epidemiologists have long enjoyed privileged access to some of the data generated by medical institutions. Diagnostic records, laboratory results and administrative datasets have been available alongside other auxiliary datasets (e.g. postal codes, demographics, population density). Public data are routinely shared to aid in surveillance, in particular to detect and contain disease outbreaks. Often these data are anonymised, or used in aggregated form and thus do not legally require the consent of individuals. In the case of infectious diseases, consent is often not required even when personal data are used in identified or identifiable form; the public interest in containing the spread of the disease is considered to outweigh personal interests in privacy or autonomy that would otherwise be protected by consent mechanisms (Lee et al., 2012). Traditionally, epidemiological research and surveillance programmes have faced many challenges, including for example slow or inadequate reporting in the case of disease epidemiology, or insufficient research programmes for pharmacovigilance (Ness et al. 2009).

Emerging digital data sources change the landscape in several respects. An unprecedented amount of data that can potentially provide insight into the health of individuals and populations is now available. Technologically, it has become much easier to aggregate and use datasets within a wide range of domains and sources including public archives, personal communication devices, and internet platforms (Salathé et al. 2012). New types of digital data generated by the interactions with networked devices and platforms can prove valuable for epidemiological purposes. In sum, going even beyond what Jacob Metcalf and others calls the characteristics of “pervasive data” - namely that not only research participants are at risk, but also people downstream who had nothing to do with the data collection; that there is no clearly bounded intervention; that the datasets themselves can be changing very rapidly; and that technology, not methodology, often determines what is done (Metcalf 2017; see also PERVADE Project, 2017) - digital epidemiology has a number of features that set it apart from biomedical research in the paper age.

This situation presents opportunities but also difficulties for epidemiology. The comprehensiveness of disease data collected from conventional sources is undermined when patients bypass formal health institutions at which diagnostic data has traditionally been collected (Prainsack 2017). People with symptoms who used to see a doctor (and thus found their way into the formal health system and through that also into formal health datasets) might instead increasingly consult online medical advice (Bovi 2003; Fox 2003; Thompson and Black 2010; Lupton 2013). Similarly, samples that used to be collected by medical staff and sent to an accredited lab for testing might now be sent directly to a lab chosen by the affected person (who would not even count as patient sensu stricto any more).^1^ Similar evidence of people bypassing established institutions can already be found in the context of research, seen for example in the rise of ‘participant-led research’, or informal research studies led by users of medical websites and patient portals (e.g. PatientsLikeMe), often using self-reported or self-collected data.

Thus, new digital data sources challenge the implied social contract between health systems, patients and epidemiologists undertaking research and disease surveillance. These sources require new contracts between data generators (often the data subject herself), data controllers and data processors regarding their contribution to the public good that has traditionally been achieved through epidemiology. Accepted principles of biomedical ethics, such as informed consent granted by individuals for well-defined, specific purposes, may prove inadequate to govern access to the ‘data exhaust’ inadvertently created through interactions with online platforms (Vayena et al., 2015a, b; Evans 2016; Prainsack & Buyx 2016).

In response to the challenges posed by emerging digital sources of health-related data, in this paper we consider whether individuals have a duty to participate in digital epidemiology, or epidemiological programmes involving individual-level data generated through online activity rather than interactions with the formal health sector. We understand the term ‘participate’ in a twofold manner (see Table 1): first, allowing existing personal digital data or information to be used for epidemiological research; and second, in terms of actively producing personal digital data or information where none is available. We examine the extent to which the implicit duty in traditional epidemiology that grants access to clinical and laboratory data should be extended to these new types of digital data. We consider in detail how such a duty would manifest in particular for users of social media platforms.

**Table 1:** Types of duties to participate

Participation Type 1: Duty to share existing data	Participation Type 2: Duty to create new data
Allowing existing digital data or information to be used for DE	Actively producing personal digital data or information for DE

The paper is structured as follows. In A duty to participate in epidemiology section we describe digital epidemiology as an evolution of traditional epidemiology that includes personal and proprietary digital data alongside formal medical datasets, and consider the risks imposed by re-purposing such data for DE. Balancing public and individual interests and A duty to share vs. a duty to create data sections then consider traditional justifications for epidemiology that imply a duty to participate for the general public, which take account of the immediacy and plausibility of threats (see: Table 2), and the identifiability of data to be processed, before distinguishing between a duty to share and duty to create. Duty to participate in digital epidemiology section then considers the case of social media data in digital epidemiology, and assesses how the duty to participate in epidemiology is transformed by online social data. Criteria to assess a possible duty to participate in digital epidemiology section proposes initial justificatory conditions for a duty for data subjects to participate in DE in the context of social media. An example: digital epidemiology with social media section demonstrates how these these conditions can be applied in practice by analysing three sample cases of social media data used for digital epidemiology. Finally, Conclusion section concludes with open questions to be considered in the public negotiation of future DE.

**Table 2:** Relative strength of the duty to participate in epidemiological research based on public health interests (insofar as existing personal data and information are concerned)

	Imminent threat	Future opportunity
Plausible benefits	Strong duty	Medium duty
Implausible benefits	Medium duty	Weak duty

## A duty to participate in epidemiology

The relationship between patients, medical professionals and the medical research community, including public health professionals, is a longstanding topic of interest in biomedical ethics (Childress et al. 2002). Medical professionals and researchers have long required privileged access to the bodies, samples and data of patients in order to learn their craft and advance medical knowledge (Pellegrino and Thomasma 1993). Negotiation and justification of this access is a longstanding issue in biomedical ethics, varying according to the purpose, aims, type of data or information involved, and the relative risks and benefits.

Informed consent is a core requirement of contemporary medical research ethics (General Medical Council 2008). Consent normally must be sought from patients prior to research studies involving access to their bodies, samples or data. Patients must be given information about the scope and purpose of data collection or access, its risks and benefits, and make an informed decision. However, access to a patient’s body, samples and data is in some cases achieved without informed consent, for example when consent is infeasible or impossible to attain, or when the link between the patient and sample or data has been broken (e.g. via de-identification or anonymisation), or when alternative compelling interests or justification can be given for performing the research without consent (e.g. provision of care, legal grounds; see below). Access in these cases is of course not granted by default; rather, valuable public or scientific interests must be demonstrated.

Access is not a one-way street, wherein patients charitably grant access to their bodies and data for the sole benefit of medical professionals and researchers. Rather, patients contribute to the advancement of medical and public health knowledge, and thus help improve the care they receive. This means that researchers must be faithful to the goal of creating public benefits, and demonstrate that they do this. On the side of patients, the emphasis on public interests has led some authors to argue for the existence of an implicit *moral* duty for individuals to participate in biomedical research by offering their time, bodies or data.^2^ In effect, a duty to participate recognises that certain public interests can, in specific well-justified cases, override the interests of individuals normally protected by informed consent.

A number of justifications can be advanced for a moral duty for patients to contribute to biomedical research. A *beneficence* argument connects the duty to participate to the individual’s capacity to prevent harm to others by contributing to medical knowledge. Beneficence-based duties can, however, quickly create overly demanding obligations for individuals, wherein individuals are expected to contribute time, resources, and accept significant risks for the sake of others (Schaefer et al., 2009). Going further, a *free-riding* argument suggests that individuals who refuse to participate are not ‘doing their share’ to contribute to a shared good; the individual enjoys medical knowledge ‘for free’, without making an in-kind contribution. As Schaefer et al. (2009) point out, “a free-rider obligation requires relief for people currently contributing”; the individual’s failure to participate is problematic in that it is unfair to others currently contributing to the shared good.

A *public goods* argument considers medical knowledge to be a public good, or one that is non-rivalrous and non-exclusive, and cannot reasonably be denied to someone requesting it. No direct incentive exist for a particular individual to contribute to medical knowledge (other than helping others or contributing to the public good), insofar as one’s benefit from it does not depend upon one’s in-kind contribution. Reflecting this, obligations are often prescribed by a society for individuals or organisations to maintain a public good, for instance through taxation. If the knowledge derived from biomedical research is treated as a public good, individuals can be compelled to participate in research due to its benefits for society and future patients (Schaefer et al., 2009). A related argument is based on the *principle of solidarity.* Chadwick & Berg (2001) argue that a “duty to facilitate research progress and to provide knowledge that could be crucial to the health of others” emerges out of the principle of solidarity. Often, research conducted without informed consent is restricted if it will “not directly benefit those participating.” Chadwick and Berg’s solidarity approach in contrast argues that societal or public benefit is sufficient to justify a duty to participate. This is not to say that the interests of individuals - symbolised by consent requirements - must be overridden, but rather that individuals can simultaneously have an interest in solidarity with others which justifies research independently of consent.^3^ This stands in contrast to Prainsack & Buyx’ work on solidarity, who argue that although policies can (and often should) highlight the public benefits created by biomedical research (if it does create them), no legal or moral duty to participate in biomedical research can be inferred from the fact that it does produce public benefits (Prainsack & Buyx 2013, 2016).

As these arguments aim at demonstrating, reasons can be advanced for a *moral* duty to participate in biomedical research. However, under existing data protection and human rights law, members of the public do not have an explicit *legal* duty to participate in biomedical research. At most, an implicit duty to participate can be observed in a limited set of cases in which the interests of individual patients protected by consent and data protection provisions are routinely overridden for public benefit.

Here, we are interested in the existence of a moral duty to participate in public health and epidemiological research in particular. In this context, many legal systems provide that secondary analyses of medical and relevant publicly available datasets can be undertaken without explicit and specific consent from patients. Three cases stand out.

In the first, pressing and serious public health interests render the compromise of patient interests proportionate (Lee et al., 2012), for instance the outbreak of an epidemic which poses a substantial public health risk. Public health is concerned with the well-being of populations (Childress et al. 2002). In public health surveillance, individual interests in privacy and autonomy are often overridden on the basis that individual data subjects benefit directly from treatments or controls on the spread of a disease, or indirectly from the scientific knowledge generated. Undertaking public health action without consent implies a hierarchy between public and individual interests. When there is a serious threat to population health—such as in light of a possible pandemic—public health measures infringe on individual rights and interests for the sake of collective interests, i.e. the health of a population.

The second case is that data are de-identified prior to being re-purposed for research. Many risks to participants in secondary analyses stem from the identifiability of data. Four types of data can be distinguished according to identifiability, the sharing of which requires increasingly strong public health interests:

- **Anonymised data** – Tracking of identifiable individuals is impossible. Identifiers have been permanently and irreversibly removed, or were never attached to the data.
- **Potentially de-anonymisable data** - Due to number of variables contained (e.g. age, geographic location etc.), re-identification may be possible through reverse engineering.
- **Pseudonymised data** - A key linking the data to an identifiable individual exists, which poses a further risk beyond re-identification through reverse engineering.
- **Identifiable data** - Linked to a unique identified individual.

De-identification is generally taken to lower the risks to participants (Ohm 2010; Kaplan 2014). However, in the era of digital data that can be linked and integrated more easily than previously, it has been argued that all data are potentially identifiable (Ohm 2010). Research with de-identified data can also pose risks to groups and populations, such as patient cohorts or profile groups (Hildebrandt 2012; Floridi 2016; Mittelstadt et al. 2016).

Given data subjects’ interests in autonomy and privacy (e.g. controlling data about oneself), the identifiability of data to be shared for an epidemiological study affects the relative risks faced by the participant. The potential risks for participants are relatively few if the data provided are collected anonymously in the first place, and do not allow for inference of identity.

However, the data needed for the public health purposes often cannot be anonymous to achieve the intended purpose. Often, data from different sources need to be linked. If the link between them is the individual (which is often the case), then the data need to be sufficiently identifiable to allow for records from different sources to be linked; pseudonymised data are required at a minimum for such linkages. Other times, the individual needs to be identifiable to those who work with the data, for instance to allow for further communication of findings or follow-up data collection. Moreover, as outlined above, even if data use in DE does not negatively affect the data subjects of the original data collected, the use of predictive analytics or profiling further downstream could affect the privacy of other people (e.g. if inferences about specific characteristics are made on the basis of other characteristics that people are not aware of, or may not have agreed to share; see Mittelstadt 2017).

A third case involves sharing of identifiable data without consent when overriding public health interests exist. Recognising the demand for identifiable data in public health research, data protection legislation in many countries already allows identifiable data to be used for public health purposes without the consent of the data subject (Gostin LO, Sapsin JW, Teret SP, et al., 2002). In Europe, the Data Protection Directive (which is still in force but set to be replaced by the General Data Protection Regulation in May 2018) allows Member States to authorise sharing of identifiable and sensitive categories of data where important overriding public (health) interests exist (comparable provisions exist in the General Data Protection Regulation). As with the other cases, determining when public health interests are sufficiently strong to override individuals’ interests normally protected by consent requires case-specific assessment.

### Balancing public and individual interests

Regardless of the prevailing justification for a study or intervention in which individual consent is not sought and a duty of participation is implied, researchers and public health authorities do not have *carte blanche* to compromise individual interests where they conflict with public health and wellbeing. Instead they must balance public and individual interests.

Striking this balance is not straightforward, as many individual interests can be violated by epidemiology (Vayena et al. 2015b). Sharing and re-use of personal data inherently carries risks for confidentiality, privacy and autonomy (Chadwick & Berg, 2001; Metcalf et al., 2017). Data subjects can be harmed by suboptimal care, distress, reputational damage, and more generally a loss of privacy resulting from misuses of data by public health professionals and researchers (Laurie et al. 2014).^4^ Systemic impacts on medical practice and the doctor-patient relationship are normally difficult to predict (Chadwick & Berg, 2001; Coeckelbergh, 2013). The balance between public and individual interests can also be tempered by choices in the design of the epidemiological intervention. Voluntary rather than mandatory reporting schemes, or reporting cases rather than identified patients can, for example, reduce violations of participant autonomy and privacy. Historical cases of mandatory reporting schemes for tuberculosis and HIV infections demonstrate how poorly designed interventions can place unnecessary burdens on individuals for the sake of public health interests (see An example: digital epidemiology with social media section; Bayer and Fairchild, 2000).

At a minimum, a guiding principle of all data provision is respect for autonomy of data subjects, which is normally reflected through the requirement of individual informed consent. Even in cases where autonomy cannot be maintained in full, the compromise of individual autonomy for collective benefit must be proportionate. As indicated above, privacy interests deserve similar consideration, at a minimum due to their position in data protection and privacy law.

Childress et al. (2002) provided one of the earliest influential formalisations of this balance between public and individual interests in public health research. Nine general moral considerations, understood as “clusters of moral concepts and norms that are variously called values, principles, or rules,” were proposed to summarise the terrain of public health ethics:

- “producing benefits;
- avoiding, preventing, and removing harms;
- producing the maximal balance of benefits over harms and other costs (often called utility);
- distributing benefits and burdens fairly (distributive justice) and ensuring public participation, including the participation of affected parties (procedural justice);
- respecting autonomous choices and actions, including liberty of action;
- protecting privacy and confidentiality;
- keeping promises and commitments;
- disclosing information as well as speaking honestly and truthfully (often grouped under transparency); and
- building and maintaining trust.”

Together, these considerations provide a basis to justify public health research and interventions that promote the public good. Childress et al. (2002) recognised that these considerations can often come into conflict, requiring ranking or weighting in specific cases where justification is sought. To aid in resolving such conflicts, five further “justificatory conditions” were derived that must be met for specific studies or interventions to be justified: (1) effectiveness, (2) proportionality, (3) necessity, (4) least infringement, and (5) public justification (Childress et al. 2002). Together, these conditions ensure that, when individual interests are violated by a public health action, the action will likely achieve public health benefits that outweigh the violated interests, using the least invasive methods available. Further, the need to violate individual interests will be publicly justified to the affected populations. Lee et al. (2012) generally reflect each of these five conditions in calling for “a well-designed surveillance system that engages affected communities, collects the minimum data necessary, stores data securely, and uses data for public health action.”

As these justificatory conditions for epidemiology suggest, the *immediacy* of a threat and *plausibility* of the anticipated public benefit are critical to the justification of a duty to participate. An appropriate balance between public and individual interests must be struck for a duty to participate to be established. But what is appropriate? The public and individual dimensions interact insofar as a duty to participate can only be recognised where an epidemiological intervention addresses a real threat, and will plausibly deliver public benefit that includes participating individuals, at least with a high likelihood. Interventions lacking on either dimensions cannot justifiably discard informed consent in the name of public interests.

Outbreak detection provides a clear example of a case where a strong duty to participate will typically be argued to exist, due to an imminent threat (a communicable disease) and a highly plausible benefit to the general public (to map the outbreak and prevent further spread of the communicable disease). A weaker duty will normally be ascertained for studies with longer-term aims or less plausible benefits, e.g. where a disease does not present an imminent threat, but its longer-term incidence can be lowered. Research into risk factors of disease across a population, for instance, has plausible benefits (identification of risk factors), but longer-term aims (to prevent - or improve future treatment of - the disease).

Generally speaking, insofar as the use of existing data or information are concerned (see Table 1, duty to share), the more immediate and tangible a threat to public health, the stronger the individual duty to participate in epidemiology becomes (see Table 1). An epidemic with high case mortality rates would thus create a strong duty to participate. Plausibility operates similarly: the more plausible the benefit of epidemiological research in responding to a perceived threat to public health interests, the stronger the duty to participate. Plausibility is determined not only by methodological validity and predicted benefits, but also by the plausibility of predicted benefits and risks to participants.

Insofar as a the active production of data or information for use of DE is concerned (see Table 1, duty to create), in general such a duty will be harder to argue for as it requires much more from individual data subjects. We will discuss this in the next section. In both cases, however, it is important to note that the existence of a duty to participate, as well as the assessment of whether it is a strong or a weak duty, must depend on an assessment of each individual case.

According to the *immediacy* and *plausibility* criteria, duties of participation may routinely be stronger for epidemiology. Responses to immediate threats are more common in infectious disease epidemiology than in biomedical research in general. This implication is reflected in the observation that reporting data is routinely shared during epidemics with public health bodies without explicit justification or public consultation.

### A duty to share vs. a duty to create data

Opportunities for data sharing between formal health systems and other public bodies on the one hand, and public health researchers and epidemiologists on the other, suggest that the ‘duty to participate’ can be interpreted in (at least) two ways:

- **Duty to share**: Individuals have an obligation to grant researchers access to data or samples about them that already exists, or to passively consent and not raise objections to secondary analyses;
- **Duty to create**: individuals have an obligation to participate in research by generating new records and samples, for instance by participating in a clinical trial or by using particular digital technologies, e.g. social media, search engines or personal health tracking devices that share data with researchers

In clinical research, a duty to participate would normally entail a duty to create, or a duty to volunteer one’s time and body for experimentation or collection of new data and samples. The duty to create can also refer to the proactive making available of records to others by a patient, seen for instance in notifying public health authorities of the contraction of a communicable and notifiable disease.

The possibility of re-using existing medical data also allows for a ‘duty to participate’ requiring less effort from the participant. The so-called ‘duty to share’ entails only that the participant allows existing data and samples to be shared with researchers, which can be achieved both with and without informed consent mechanisms in place. As Lee et al. (2012) have argued, ‘participation’ in the context of epidemiology equates to, at a minimum, data sharing without *explicit* and *specific* individual consent. In other words, if a duty to share is recognised, patients should allow records generated through their interactions with the formal health sector (e.g. diagnostic or administrative records) to be shared with epidemiologists by default, so long as minimally sufficient protections (e.g. technical and organisational security, data protection measures) are in place.

As this suggests, the duty to share can be fulfilled passively, for instance by patient groups not objecting to agreements between data controllers and public health authorities or researchers. Here, we are considering an individual duty to share on the side of data subjects; however, it is also feasible to consider a duty to share for third party data controllers (but we will not do so here). Users of platforms that generate relevant data and contributors to relevant public datasets can similarly choose not to raise objections to data sharing with researchers.

In practice, the two duties will often overlap in a single ‘duty to participate’; clinical trials can, for instance, require access to participants’ existing medical records. Assuming the methods, aims and purposes of an action are equivalent, justifying a duty to create would normally require stronger public interests than the duty to share. This is due to the greater effort required by participants and risks stemming specifically from data collection. Further, a duty to create would imply a duty to share the data with the body that requested its creation. Additional duties to share the created data with other parties can also be recognised separately.

A programme undertaken by the Chicago Department of Public Health shows both duties in practice outside of a formal health system, and demonstrates the additional burdens imposed by a duty to create. The ‘Foodborne Chicago’ programme was launched in 2013 aiming at improving the city’s food safety.^5^ Foodborne Chicago accesses and mines Twitter data for phrases or words referring to food poisoning. Algorithms comb through the data to identify relevant tweets. Humans then verify their relevance to food borne diseases and determine whether a tweet merits a response by the programme. The tweets that are considered most likely to refer to food poisoning receive a reply (via Twitter) inviting the person to file a report of their food poisoning incident. Initial data show that from the thousands of tweets flagged by the algorithms, 12% received a reply. On the basis of these reports the Department of Health initiates inspections, and it has reported that at least 40% of these inspections revealed violations of the health code. In this case Twitter users’ data are mined by Foodborne Chicago, without users’ explicit consent.

In this case a duty to share is reflected in the department’s analysis of Twitter data without seeking individual consent, or instigating public debate about the legitimacy of such uses. The justification for the use could be either that the tweets are publicly available information, or that the legal agreement between a proprietary platform and its users (‘Terms and Conditions’) may explicitly allow for such uses. A moral duty to share is implied regardless of the availability of data or legal agreements in place in the mere fact that consent is not being sought; permissive legal frameworks can themselves imply a duty to share by allowing for agreements that permissively allow sharing of data with third parties for research.

Concerning the duty to create, for a full reporting, users are only invited and not obliged to report. Although an expectation currently exists that the privacy and liberties of patients with notifiable diseases can be infringed for the sake of public health, an equivalent standard does not exist for individuals with foodborne illnesses. Therefore they are not obliged to report their incident despite the fact that a restaurant can be responsible for an outbreak with serious consequences for public health. A duty to create data is thus not explicitly enforced, insofar as authors of relevant Tweets are invited rather than compelled to reply and file a report.

## Duty to participate in digital epidemiology

As the preceding discussion suggests, an implicit duty to share, understood as a type of duty to participate, can be recognised in epidemiology when interests in public health or the advancement of medical knowledge override the interests of individuals normally protected by consent. It may not be possible, however, to simply extend this duty to cases where epidemiology involves new, non-medical digital data sources—what we have termed ‘digital epidemiology’. It is unclear whether the underlying social contract remains valid in the case of digital epidemiology. To examine the validity of extending a duty to participate to DE, it is necessary to examine the differences between digital and traditional epidemiology.

We distinguish *digital* epidemiology according to the *routine* processing of data that describe (1) health or are health-related (what we term *non-medical*)*,* meaning they have not been sourced from a formal medical institution or service but can be used to generate knowledge about health^6^; are (2) *personal* and granular, meaning they describe the behaviours and health of individuals (even if the individual is not identifiable)^7^; and are (3) *proprietary* or private rather than public, meaning they have been created through interactions with proprietary online platforms and technologies that offer limited public accessibility to the data collected. The proposed characteristics are intended to be indicative rather than exhaustive to provide a working definition of DE. The presence of any of the three characteristics in a study suggests that it may be a digital rather than traditional epidemiological study. As this suggests, the line between digital and traditional epidemiology is imprecise.

According to this definition, DE is distinct insofar as it involves *routine* processing of granular personal data (as opposed to population-level data) that form a record of an individual’s behaviour, for instance their interactions with online platforms, services and devices. The term ‘routine’ emphasises that DE commonly involves proprietary, digital datasets describing the behaviours of individuals in detail, or at least more frequently than has historically been the case in epidemiology. These data are generated outside of formal medical or public health institutions, from personal domains in which each data contributor is unique. Much of the data used in DE is not strictly speaking medical data, or sourced from formal healthcare systems, but rather (commercial) data generated for purposes unrelated to health which can be re-purposed for epidemiology.^8^ This is not to say DE data are not about health, but rather that the original purpose of their collection is often not for formal medical purposes^9^; the data are nonetheless valuable to DE insofar as inferences about the health of individuals or related relevant parameters can be drawn. These data will further often be private or proprietary, with access requiring negotiation with a commercial platform or service provider. Recognising these unique characteristics of the data used in DE, we adopt a sectoral approach to defining DE, according to which the source of data and purpose of processing determines whether a study can be considered digital epidemiology or traditional epidemiology.^10^ This approach highlights that DE routinely involves data generated outside of formal healthcare systems or public health surveillance.

The types of data used in DE may pose different risks to participants than those associated with uses of formal medical and public health data. As described above (see A duty to participate in epidemiology section), processing of any personal data poses risks for the privacy and autonomy interests of individuals. Compared to striking a balance between public and individual interests in traditional epidemiology as discussed above (see Balancing public and individual interests section), DE adds a further layer of complexity including personal data that do not directly describe health or medical measurements, but rather health-related behaviours or relevant contextual information. On this basis the justification of a duty to participate must be re-considered.

Three differences in the risks posed by DE stand out. First, the potential invasiveness of data under consideration is routinely higher, as the data considered in DE describe the behaviours and characteristics of an individual over time, rather than those of a population. Participants grant a more granular and personally revealing view of their activities than would normally be the case for population or group level datasets. Second, data need not strictly describe health parameters to be used in DE. Participants in DE thus risk exposing aspects of their lives unrelated to health. Third, DE researchers can routinely negotiate access to a data subject’s proprietary records without involving the data subject or asking for consent. Given the proliferation of personal data in mature information societies, greater effort is required from participants to track and control how their data are used in DE. The autonomy of data subjects is therefore weakened, insofar as control of personal data is undermined. The risks imposed by DE thus go beyond potential harms related to the disclosure of health status or medical history, and include more general ethical risks of internet research (Markham et al. 2012) and Big Data analytics (Mittelstadt and Floridi 2016).

An individual’s expectations of privacy and expected uses of their data may also differ when data are generated through interactions with proprietary platforms and services, as opposed to formal health services. DE may thus pose different or more severe privacy risks due to the data types taken into consideration, which describes individuals rather than populations. This is not to say such data cannot be aggregated, or that population-level data generated by new digital sources cannot be used in DE. Rather, it is the particular availability of individual-level non-medical data that disrupts the balance between individual and public health interests grounding the duty to participate in epidemiology as discussed above (see A duty to participate in epidemiology section).

## Criteria to assess a possible duty to participate in digital epidemiology

As with the duty to participate in biomedical research, ‘justificatory conditions’ can be identified for a duty to participate in DE that, if met, suggest an appropriate balance has been struck between public and individual interests, and relevant general moral considerations. As with public health research, digital epidemiology pursues promotion of public health and the advancement of medical knowledge. Recognising this, the nine general moral considerations and five justificatory conditions for public health ethics, defined by Childress et al. (2002) (see A duty to share vs. a duty to create data section), provide a logical starting point to assess whether a duty to participate in digital epidemiology exists.

Here, we define a set of eight justificatory conditions for a duty to participate in DE conducted by public health bodies and researchers.^11^ To define this set, the authors considered the relevance of the general moral considerations and justificatory conditions specified by Childress et al. (2002), and how their application is affected by the new types of data and risks involved in digital epidemiology. In defining such conditions, the unique risks posed by DE, which vary according to the type and source of data and the purposes of processing, needed to be addressed. Contemporary work on public health ethics reviewed in Balancing public and individual interests and A duty to share vs. a duty to create data section was also considered. Following this initial analysis, three cases of digital epidemiology were defined and analysed (see An example: digital epidemiology with social media section).

The set is intended to provide a structure for consideration of concerns in specific cases of digital epidemiology. The set is not designed to serve as a ‘checklist’, or as a threshold to establish the existence of a duty in a particular case. The conditions provide a list of concerns to guide a structured discussion in a particular case whether a duty to participate can be justified.^12^ As proposed above, the *immediacy* and *plausibility* of a proposed DE intervention influence the relative strength of the duty to participate, and the precise balance required between individual and public interests for a duty to participate to be established. The justificatory conditions proposed here are minimal conditions to be met by DE, regardless of relative immediacy and plausibility (see Balancing public and individual interests section). We demonstrate how this assessment can proceed through the cases analysed in An example: digital epidemiology with social media section.

As discussed above (see Balancing public and individual interests section), general moral considerations and justificatory conditions for public health research and interventions can conflict and will need to be weighted or prioritised differently in different cases (Childress et al. 2002). In specifying eight justificatory conditions for digital epidemiology, we do not recommend a particular weighting or prioritisation to resolve such conflicts; nor do we suggest that a study meeting a minimal number of the conditions will necessarily be justified. We do not, for example, argue that potential improvements to patient safety achieved through digital epidemiology always outweigh individual interests in privacy.^13^ The relative importance of public and individual interests, and the eight justificatory conditions reflecting them, can only be decided on a case-by-case basis (Childress et al. 2002), with due consideration of local interests and risks.

1. **There is a strong public interest in disease prevention**
  Digital epidemiology actions must be driven by public interests in the advancement of medicine or public health (Childress et al. 2002). These interests do not need to be specific or in response to a particular event, such as the outbreak of a contagious disease. Example public interests include emergency containment of an outbreak, identification of at-risk populations for preventative measures, and general advancement of medical knowledge about the contributing factors and treatment of a health condition. Relevant interests driving DE for which a duty to participate can be established are therefore limited to public interests, meaning commercial or private interests would not be sufficient. This condition limits the types of interests that can be appealed to in order to justifiably violate individual interests normally protected by consent. DE aimed at targeted advertising of pharmaceutical products would, for instance, fail to serve a valid public interest.
2. **It is plausible to assume that the use of a person’s data will contribute to disease prevention**
  As suggested by discussion of *plausibility* above (see Balancing public and individual interests section), for a duty to participate to be valid, it must be assumed that the methods involved are sound, and will thus produce a benefit to public health or medical knowledge. This condition is similar to the *effectiveness* condition proposed by Childress et al. (2002) in their discussion of public health ethics. The validity of DE methods cannot be taken for granted (Vayena et al., 2015a, b). However, it may prove difficult to validate particular methods prior to access being granted to the data requested, particularly for DE searching for unforeseen correlations across aggregated datasets. Application of this condition must therefore take account of the purpose of the proposed DE action; a duty to participate in exploratory DE not addressing an imminent threat but pursuing important public health interests may be justifiable when minimal risks exist for participants.
3. **The risk for data subjects are minimal, and are not significantly greater than non-participation**
  For a duty to participate to be established, the usage of personal data in DE should not pose significantly greater risks to data subjects than the non-use of such data. Application of this condition requires consideration of the level of identifiability of data involved. Where identifiable and pseudonymised data are used, appropriate security and confidentiality measures must be in place to protect participants’ interests. Such measures can include, for example, a prohibition of individual-level actions based on knowledge derived from DE unless the data subject has subsequently consented to such uses. Incidental findings or risk estimates should, for instance, not be entered into an individual’s medical record without consent. This condition roughly follows the *necessity* and *lease infringement* conditions set by Childress et al. (2002), according to which a public health action should not be undertaken if an alternative action is available that will accomplish the same results with lesser violations of individuals’ interests.
4. **There is no or only negligible effort required from the data originator**
  Data sharing for DE should not significantly disrupt the normal lives of participants. For a duty to share, minimal if any effort is likely to be required on the part of the data subject to share the relevant data. Data controllers or platform providers will normally provide access to the data in question, and thus fulfil the participant’s duty to share. However, platforms that grant users control over their data may require action from data subjects’ to share their data with DE researchers. In such cases, the effort required to do so should be minimal. If a duty to share is established, meaning consent mechanisms would not be used, effort is unlikely to be required from data subjects. For a duty to create, the actions required by data subjects to generate new data for DE should be negligible (the threshold for ‘negligible’ will vary between contexts). Ideally, existing users of relevant platforms will be engaged in the first instance. Taking the Chicago Foodborne case above, if a duty to create were established, the minimal effort condition would suggest existing Twitter users should be targeted for participation in the first instance to minimize effort required from participants (e.g. to learn how to use a new platform, or to sign up for an account that itself may prove privacy invasive). Existing users could be encouraged to send Tweets about experiences with Chicago restaurants to the programme’s account.
5. **The data subject has a broad understanding of the public health purposes for which the data will be used**
  When informed consent is infeasible, the autonomy of the data subject must nonetheless be respected. Ensuring data subjects are aware of the potential value of the data they create is therefore critical. Data subjects should ideally be informed at the point of creation (e.g. when agreeing to the terms of service of a social media platform) of potential foreseeable uses of their data for DE. While this may not always be feasible, as the potential value of data is often unclear until a particular need arises or a link to another dataset is established, data subjects should nonetheless be made aware when future research applications are foreseen, and about the general possibility of data re-purposing for DE. The existence of such broad understanding should be demonstrated prior to a duty to participate being established. If infeasible, potential participants will ideally be notified prior to the study commencing. This condition implies that platform providers have a duty to notify users of the intention to share data with third parties for DE purposes.
6. **The minimal amount of identifiable data necessary is being used**
  Following a core principle of data protection law, the minimal amount of identifiable data required should be used in DE. Data minimization contributes to the protection of privacy and other individual interests by limiting duplication or storage of irrelevant data. Although DE data will ideally be kept under secure conditions to ensure confidentiality, the latent privacy risk of data storage cannot be dismissed. To minimize risks of re-identification, data lacking explicit identifiers should be used whenever possible to minimize privacy risks to data subjects. Latent risks for group privacy must be considered, even when anonymised and population-level data are used (Taylor et al., 2017; Mittelstadt 2017). Usage of pseudonymised and identifiable data should be justified by methodological necessity, or the necessity of tracking, re-contacting or otherwise following up with an identifiable individual over time.
7. **Where there is a risk of stigmatisation of, and possible harms to, participants or participant networks, there has been engagement with the affected community to assess the risks of participation without consent**
  Health conditions and predispositions are often linked to social and other forms of stigma. Where DE can potentially reveal a link between a condition or disease and another attribute, potentially affected communities should first be engaged to assess whether an action can justifiably be undertaken without consent. As the vulnerability or potential stigma attributed to a disease increases, so too does the researcher’s obligation to engage with the affected community and protect its interests (Markham et al. 2012).
8. **Harm mitigation strategies are in place in the case that harm befalls participants and participant networks**
  Even when substantial precautions are taken, data processing can result in harm to the data subject. The Chicago Foodborne programme shows, for example, how financial loss can result from businesses that are reported via social media and come to the Department of Public Health’s attention. Without consent mechanisms, participants are not given an opportunity to assess the potential risks of participation. To fill this gap, mitigation and redress mechanisms should be in place to compensate affected participants’ and participant networks’ interests where harms arise or are expected (Vayena, 2015; Prainsack & Buyx 2013, 2016).

### An example: Digital epidemiology with social media

To demonstrate how the conditions can be enacted in a particular type of DE, we will consider several hypothetical cases that show how social media platforms can be used for digital epidemiology. There is a growing interest in such data in epidemiology (Salathé et al. 2012; Young, Rivers, and Lewis, 2014; G. Eysenbach 2008; Gunther Eysenbach 2009; McKee 2013; Velasco et al. 2014; Brownstein, Freifeld, and Madoff, 2009; Mordini 2013). Social media presents an ideal case to evaluate a duty to participate in DE, insofar as it involves proprietary non-medical data that are not explicitly created for public health purposes, and for which consent to secondary uses is largely infeasible (Markham et al. 2012; Varnhagen et al. 2005). Each of the following cases demonstrates challenges with applying the eight proposed conditions.

**Table:** 

**Case 1: Infectious disease surveillance via social media and search** *(Conditions 1–4)* Web-based platforms and digital social media have proliferated very widely in the recent decade, especially, but not only, in high-income countries. In 2015, 84% of all adults in the United States (Perrin & Duggan, 2015), and almost 40% of all Indians (Press Trust of India, 2015), used the Internet. Internet usage in low income countries is rapidly growing (World Bank 2017). Many people post information on their daily lives, including their health and illnesses, on social media platforms, or they use search engines to look up symptoms. This has been seen to provide epidemiology with a very powerful new data source to predict disease outbreaks, for instance by assuming that the geographical clustering of certain terms (e.g. “joint pain”, “fever”) can indicate high rates of contagion in a region. If used successfully it could help prevent or mitigate disease outbreaks and thus avoid pain, suffering, and significant cost to individuals and the public alike. Although some of these hopes and expectations have been found to be exaggerated - e.g. Google Flu Trends failed to predict the 2013 flu outbreak (Lazer et al., 2014) - the issues are mostly seen as methodological, and solvable. An early noteworthy example of DE was provided by Google through their Flu Trends programme, which predicted flu activity in 25 countries from search patterns linked to traditional disease surveillance data from public health institutions, such as the Center for Disease Control in the United States. Although the programme has since been abandoned (Lazer et al., 2014), other projects have continued with estimates of flu activity based in part upon data generated on social media platforms. Sickweather, for example, analyses scraped social media data to geographically map illness (Sickweather, 2016). Users can view anonymised reports of illness from social media down to street level, and be alerted when infectious disease outbreaks occur nearby. HealthMap provides similar services for individual users and public organisations, including tracking of flu and Ebola outbreaks (HealthMap, 2016). Google has recently engaged in similar work around the Zika virus to predict and visualize outbreaks of the disease based on weather, travel and other disease data (Google, 2016).	

Assuming that data from social media and search platforms can be used for infectious disease surveillance, the question remains as to whether people have a duty to (a) allow their data from being used for disease prediction purposes, and (b) to proactively enter information into social media platforms or search engines that collate information for disease prediction purposes. It is worth mentioning here that at least legally, option (a) seems unproblematic because by making use of the respective sites and platforms, data controllers already have the right to use, and allow third parties to use, user data when certain conditions are met (normally including de-identification). But is there a moral duty for users to allow their data to be used for this purpose?

To make this determination, it is necessary to know whether the eight justificatory conditions specified above are met. This can be assessed only on a case-by-case basis. However, we cannot find a reason why it would not be possible to meet each of the eight conditions in studies seeking to share pre-existing social media and search data. We therefore conclude that a duty to share is plausible in general for infectious disease surveillance via social media and search.

This leaves a duty to create, which would require citizens to actively enter such data on social media and search platforms when they would otherwise not do so. In other words, if a person has good Internet access and suspects that she experiences symptoms related to an infectious disease, does she have a duty to log on and enter these terms in a search engine, also disclosing geo-location data, or post about them on social media?

We argue that there is no such duty for people who do not routinely use the platforms and tools that are used to collect these data. Using the conditions developed above, even if there was a strong public interest in disease prevention (Condition 1) and it could be plausibly argued that the use of a person’s data will contribute to disease prevention (Condition 2), the third and fourth conditions may nonetheless not be met. If a person does not use social media or search engines the subjectively perceived risk - e.g. for her privacy to be infringed, or having to worry about the possibility of a breach of privacy - for her may increase significantly. And taking the step of using a tool that she would not otherwise use represents significant additional efforts.

For people who do routinely use the tools that data for disease prevention would be drawn from, a duty to create is more plausible. However, reporting symptoms may constitute additional effort that would otherwise not have been undertaken (Condition 3). Whether this additional effort is justifiable is context-specific. However, reporting symptoms via social media in particular carried additional risk of stigma when the created content is publicly visible. Such cases would arguably present additional risk to the participant, and thus violate condition 4. We therefore conclude that a duty to create cannot be taken for granted, but rather requires careful balancing of public and user interests to justify in specific cases. A very strong public interest would be required; our intuition is that a duty to create would only be feasible in cases of epidemics presenting substantial immediate risk to public health.

**Table:** 

**Case 2: HIV screening via social media** *(Conditions 3 and 7)* HIV/AIDS has become a distinctive case in the history of epidemiology as the disclosure of infection is widely considered to put seropositive individuals at risk of stigmatization and violence. The WHO has particularly emphasized the preservation of individual human rights against a strong public health interest, as the stigma of HIV “threatened to drive infected persons to conceal their status” (Fee and Parry 2008). With the advent of new prevention measures such as PrEP (pre-exposure prophylaxis), the widespread availability of rapid test kits and an increasing presence of risk groups on social media, DE has been suggested to solve some of the newly emerging problems in HIV surveillance and prevention. To this end studies have already tried to determine implicated health-related attitudes and behaviours on Twitter in order to fine-tune the coordinates of intervention (Young et al., 2014). Furthermore, DE is particularly praised to provide new ways of increasing the quality of surveillance data on MSM (Men who have sex with men), as traditional public health strategies seem to be failing (Young, 2015).	

The history of AIDS provides a clear case, in which the confidentiality of serostatus has outweighed public health benefits of coercive testing or legal instruments making individuals reveal their status. Even if there is a strong public interest in disease prevention through HIV screening, a duty to disclose (i.e. a duty to create) an HIV status has historically been ruled out due to the risks of stigma and discrimination. As in the case of infectious disease surveillance via social media, no duty to create can be assumed due to the violation of Condition 3; the risks of stigma to participating individuals are not negligible.

However, it has been suggested that social media data can be used to identify people who are at high risk of HIV (Young et al., 2014). This type of DE action may be eligible for a duty to share. To justify a duty to share in this context, two questions are key. If screening methods are used to identify people at risk due to the isolation of digital reflections of risk behaviour, how can it (a) be guaranteed that the same measures are not used to identify persons at risk and to expose them to stigmatization and discrimination? And (b), assuming such adverse effects against the background of AIDS history, how can screening risk behaviour prevent people from interrupting their practice of discussing and disclosing their sexual practices online? Here Condition 7 suggests substantial engagement with potential participants and participant networks is required to meet the obligation of minimizing the risk of public exposure and identification, and to discuss possible venues of active anonymised data generation if a duty to create is nonetheless pursued.

**Table:** 

**Case 3: Notifiable diseases in livestock** *(Conditions 3 and 8)* Animal health and disease are also relevant to digital epidemiology, if only for the link with human health. Farmers are already obliged to report notifiable diseases that have affected their livestock to public health authorities. Such policies aim to prevent spread of disease between animals, from animals to humans and from humans to animals. Resulting measures to control outbreaks in farms may have severe financial consequences for farmers. Compensation schemes often apply to remedy at least partly the damages resulting from disease control measures. Compensation can be seen as an incentive for reporting, since businesses can meet their public health requirements without fear of financial ruin. Beyond the financial damages, however, farmers and even entire regions may also bear reputational damages depending on the disease, publicity and success, or failure, in controlling the outbreak. Some evidence indicates that at least specific notifiable diseases may remain under-reported. For example, bovine abortions should be reported in France to detect brucellosis, a disease eliminated in France but still reporting is required. However, data show that only a third of the detected abortions are reported (Bronner et al. 2014). Social media can provide methods for detecting both notifiable and non-notifiable diseases. Farmers may reveal concerns over both notifiable and non-notifiable diseases over social media, particularly before having enough evidence to confirm a problem exists. Digital disease surveillance can pick up such signals that may lead to proactive measures on the part of health authorities. While beneficial to public health, farmers who, for example, are identified or followed for having reported concerns via social media may be subjected to additional inspections, reputational and financial damages.	

The question here is whether the farmers have a duty to create over and above what is required from them legally in the context of notifiable disease to report concerns via social media. Given that social media can instantly reach large amounts of people, possible reputational damages for a farmer who reports online may be catastrophic, even more so than pursuing the more formal channels of reporting through health departments. A duty to share can also be considered; public health authorities could routinely monitor social media accounts of relevant farmers for indications of notifiable and non-notifiable diseases.

For both a duty to share and create, condition 3 is particularly relevant. Evidence indicates that one of the concerns that farmers have with reporting in general is the cost-benefit analysis which is often unfavorable for them. If the public health benefit is significant (and the question here is how significant), can it take priority over the personal costs specified above? Condition 3 would be challenged in this case, because the risk for data subjects (in this case the farmers) can potentially be high. Condition 8 would therefore also be essential, given that compensation schemes for traditional reporting already exist. For either duty to be justified, it would appear at a minimum that significant compensation be available to affected farmers due to the financial and reputational risks of both reporting via social media, and passive surveillance of social media by public health authorities. Condition 5 would also be relevant in both cases, insofar as farmers may feel deceived if routine surveillance were in place without notification of the potential usage of social media postings for livestock disease monitoring.

## Conclusion

In this paper we have examined the ethical justification and feasibility of establishing a duty to participate in digital epidemiology, following from comparable duties in the context of epidemiology and biomedical research. We have proposed eight justificatory conditions which, if met, suggest an appropriate balance is struck between individual and public health interests (where they conflict). As the preceding discussion has demonstrated, application of the eight proposed justificatory conditions for a duty to participate in digital epidemiology requires case-specific consideration.

To begin to unpack the difficulties of applying and balancing the eight proposed justificatory conditions, we have discussed three hypothetical cases involving the usage of social media data for digital epidemiological purposes. The proposed set of conditions will face other challenges and may require expansion when applied to other non-medical, personal or proprietary data types used in digital epidemiology, for instance data generated by wearable health monitors (Mittelstadt et al. 2014) or the Internet of Things (Pasluosta et al. 2015). As the concept of health data expands (Vayena and Gasser, 2016; Vayena et al., 2016; World Health Organization 2017), further research is required to begin to understand the potential extent and force of a duty to participate in digital epidemiology across different sectors.

It may also be necessary to consider the duty to participate applying to other actors involving in the collection, processing and storage of relevant personal data. The duty to participate described here addresses the obligations and interests of data subjects. This is not, however, the only possibility: a duty to share may also exist for data controllers. These duties may in fact conflict; an individual data subjects’ autonomy could be justifiably violated due to overwhelming public health interests, while these same interests may not be sufficient to violate a data processors’ commercial interests (Vayena et al., 2015a, b). An internet platform provider may, for instance, have an interest in not sharing data due to potential reputation damage (e.g. if the data leads to invasive or embarrassing findings about their users), or due to competitive interests; the data could, for instance, allow for reverse engineering of proprietary software or the identity/demographics of the platform’s users (Mittelstadt et al. 2016). Appeals to override their commercial interests in reputation and secrecy must be made on different grounds.

This does not, however, need to pose a problem. It can be argued that data controllers have a social contract with data subjects and the societies in which they operate. The interests of data subjects can be transferred to their data controllers on the basis of the contract,^14^ meaning compelling public health interests sufficiently strong to override individual’s interests in privacy and autonomy can, by default, be taken to override commercial interests in keeping data secretive. Whether this line of reasoning is satisfactory depends largely upon the legitimacy of risks to data controllers. Datasets will pose variable risks of re-identification or reverse engineering of proprietary software (Zarsky, 2013). These risks that can only be assessed on a case-by-case basis.

In any case, a potential conflict can be seen between individuals’ moral duty to participate, and commercial interests in proprietary data (which may be legally protected). Although moral duties are not directly enforceable, individuals may increasingly be able to force controllers to share their data (in Europe) through enforcement of new rights granted in the EU General Data Protection Regulation. Specifically, the data subject’s right of access (Article 15) and right to data portability (Article 20) may provide a legal mechanism for data subjects to exercise a moral duty to participate by obtaining a copy of their data which can be shared with digital epidemiological bodies. Further research is required to establish under which conditions, and in which cases, both a moral and legal duty to share exists both directly and by proxy for third party data controllers.

Even where a duty to participate in digital epidemiology is recognised, data subjects should always in principle retain the capacity to refuse to participate. Legal coercion should only be used in abnormal circumstances presenting an immediate and overwhelming threat to public health. In practice, this would mean the data subject can specifically request that her data not be shared (possibly when agreeing to use a data generating service or platform), or refuse to use a particular platform to create new data for DE, even when moral duty to create is recognised. Beyond respecting the justificatory conditions proposed here, preserving this key consideration is crucial to responsible and publicly beneficial usage of digital epidemiology methods going forward.

## Footnotes

1 This new form of interaction enabled by the greater availability of information and direct-to-consumer medical services introduces a new way to be a ‘patient’, but outside of traditional medical institutions and services. Analysis of the implications of this new type of patient for research ethics and health services are beyond the scope of this paper.

2 It is important to note that moral and legal duties are not equivalent. For our purposes, we can consider a *moral* duty to be a voluntary duty, whereas non-compliance with a *legal* duty will have legal effects on the data subject. For a related discussion of moral and legal *rights* and duties that flow from them, see Jones (2016).

3 Solidarity cannot, however, be taken for granted as a prevailing value in a population. Chadwick & Berg (2001) note that sufficient protections must be in place for data subjects to be expected to value solidarity, and accept alternative broad consent mechanisms: “It is increasingly recognized that an informed consent process that is lengthy and complex might be more burdensome than protective[…]Individuals need to be protected and respected, and there needs to be adequate data protection to safeguard against misuse, discrimination and stigmatization. It is arguably neither morally nor practically feasible to expect individuals to show solidarity in the absence of such protections.”

4 According to Laurie et al. (2014), misuses include fabrication or falsification, theft, unauthorised disclosure, non-secure disposal and excessive retention of data.

5 See http://www.foodbornechicago.org/pages/about

6 We distinguish here between ‘medical data’, ‘health data’ and ‘health-related data’. ‘Medical data’ describes data from formal medical sources, or produced by formal healthcare systems. ‘Health data’ describes a health parameter (e.g. heart rate), but is not produced within a formal medical system. ‘Health-related data’ does not describe a health parameter, but can be used, often in combination with other data types, to infer knowledge about health.

7 The usage of the term ‘personal data’ here runs somewhat counter to current European data protection law under the 1995 Data Protection Directive (95/46/EC), according to which personal data must be linked to an identified individual. Here, ‘personal data’ refers to data describing a unique individual, regardless of whether they are identified. We are thus interested in personal data that allows for an individual’s records to be linked over time and, in some cases, across different platforms and datasets. This approach follows the forthcoming EU General Data Protection Regulation’s inclusion of pseudonymised data (i.e. data that is linkable in principle to an identified individual) as a type of personal data.

8 Data generated by personal health monitoring devices (Mittelstadt et al., 2014), for example, can record physiological parameters for commercial well-being or fitness monitoring in the first instance, as opposed to formal medical care.

9 It can be countered that digital epidemiology is not different from traditional epidemiology according to definition. Epidemiology has a long history of re-purposing non-medical datasets for medical purposes, including data on public housing, population density, animal density, sea traffic, climate and postal codes. This counter argument is, however, somewhat misleading. While each of these data types can describe populations or individuals, they differ in temporal scope; DE involves datasets that are routinely richer in intervals or events captured describing individual behaviour. In other words, individual-level data is DE is normally longitudinal and sometimes continuous, insofar as a record of the individual’s behaviour over time (e.g. a history of Tweets) is used, as opposed to a record of the individual’s status at a single (or few) point in time. Further, the need to negotiate access to datasets generated via proprietary, digital platforms distinguishes DE from usage of such public datasets in traditional epidemiology. This is not to say DE will not use public datasets, or that traditional epidemiology never involved proprietary data, but rather that the routine consideration of proprietary datasets is unique. The issues encountered with negotiating access to clinical and public datasets in epidemiology in general remain applicable to digital epidemiology.

10 This approach reflects the ambiguous definition of ‘health data’ or ‘health-related data’, seen for instance in the forthcoming EU General Data Protection Regulation. The approach taken in the Regulation is to classify data as health-related or not (and thus, sensitive or not) based solely upon the purposes of processing. The sectoral approach described here is roughly similar, insofar as it is a combination of the question being asked and the fact that data has not come from a formal medical source that allows a study to be classified as DE. It follows from defining ‘health-related data’ in this way that it users cannot know when creating data whether knowledge about health can be inferred from it, and therefore whether it can be used in digital epidemiology. The scope of work considered to be *digital* epidemiology is therefore much broader than traditional epidemiology.

11 As noted above, in many cases, the relevant data for digital epidemiology will be sourced from commercial data controllers and platforms. Our intention is not to define justificatory conditions for commercial data controllers, but rather for public health authorities and researchers that have managed to obtain access to this data through negotiation with data controllers, scraping publicly available data, or open access datasets. Commercial data controllers may in fact perform analyses comparable to digital epidemiology; however, this type of application (e.g. Google Flu Trends programme) is not normally considered to be within the scope of biomedical or public health research ethics. Justificatory conditions for commercial ‘digital epidemiology’ is an interesting, but distinct problem outside the scope of this paper. We limit our consideration to the existence of a duty to participate in bona fide medical research and public health surveillance, rather than a duty to participate in internal analyses performed by commercial data controllers.

12 Additionally, we do not engage with the question of tradeoffs between the benefits (in terms of higher ethical standards) and costs (financial or otherwise) of application of the justificatory conditions in particular cases. In data-based research and practice, higher ethical standards are often accompanied by greater costs. For example, the costs of re-contacting participants for secondary uses of their data can quickly prove prohibitive in large datasets containing data from thousands or millions of individuals. Determining when such costs are appropriate, and an appropriate balance between protecting individuals’ interests and the costs on data controllers of doing so, must similarly be determined in assessment of particular cases of digital epidemiology.

13 However, we recognise that digital epidemiology researchers create significant value through their analysis of new, personal non-medical data types; their interests in protecting and applying the knowledge generated must also be protected (Chadwick and Berg 2001).

14 This type of social contract should not be confused with data controllers acting as a proxy to fulfil the data subject’s duty to share; large volumes of data would normally be shared with researchers by third party data controllers, not individual data subjects.
